# Analytical Application of an l‑Cysteine-Based Electrochemical Sensor for Quetiapine Determination in Pharmaceuticals

**DOI:** 10.1021/acsomega.5c07368

**Published:** 2025-09-30

**Authors:** Maria Eduarda C. Goulart, Lucas D. Paquini, Luciene P. R. Profeti, Demetrius Profeti, Bruno R. L. Ferraz

**Affiliations:** a Department of Pharmacy and Nutrition, Federal University of Espírito Santo-UFES, 29500-000 Alegre, ES, Brazil; b Laboratório de Pesquisa e Desenvolvimento em Eletroquímica, Federal University of Espírito Santo-UFES, 29075-910 Vitória, ES, Brazil; c Department of Chemistry and Physics, Federal University of Espírito Santo-UFES, 29500-000 Alegre, ES, Brazil; d Department of Biology, Federal University of Espírito Santo-UFES, 29500-000 Alegre, ES, Brazil

## Abstract

The electropolymerization of l-cysteine was applied for the development of sensors to determine quetiapine in tablets. The proposed sensor was characterized using electrochemical impedance spectroscopy and applicability in the analysis of quetiapine by square wave voltammetry. The electrocatalytic oxidation and pH effect of quetiapine using the sensor were investigated through cyclic voltammetry. The voltammetric peak currents, measured under optimized conditions, exhibited linearity over the quetiapine concentration range of 8.05–85.0 μmol L^–^^1^, with a detection limit of 1.17 μmol L^–^^1^. The proposed poly­(l-cys)/GCE-based electrochemical sensor exhibited successful applicability in the selective quantification of quetiapine within pharmaceutical samples.

## Graphical abstract

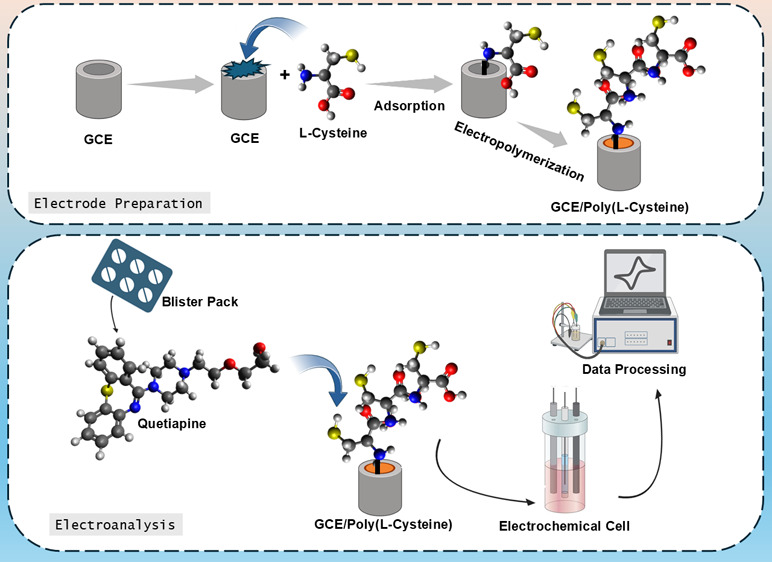

## 1 Introduction

Quetiapine (QTP), a dibenzothiazepine-class atypical antipsychotic, is widely prescribed for the treatment of psychiatric disorders such as schizophrenia, bipolar disorder, and depression that are unresponsive to conventional therapies. Its mechanism of action involves antagonism at the dopamine D_2_ and serotonin 5-HT_2_A receptors. In the low dosage, it is used for insomnia treatment while at moderate and high dosages is used for humor disorders and schizophrenia, respectively.^1^ The active principles analysis is an essential aspect of pharmaceutical quality control and pharmacokinetic and bioequivalence studies during all pharmaceutical development.^2^

Psychoactive drugs found in aqueous effluents pose a threat to the aquatic ecosystem as they alter the behavior and physiological functions of organisms, compromising their reproduction and survival.^3^ Accordingly, the development of straightforward, highly sensitive, rapid, and reproducible analytical methodologies is of great relevance. For both bulk substances and finished pharmaceutical formulations, an analytical method must be capable of simultaneously detecting the parent compound, its related impurities, and degradation products. Moreover, all quality control procedures must be conducted in strict compliance with current pharmaceutical regulatory standards.^4^ Various analytical methods have been developed for QTP quantification in pharmaceutical and biological samples, such as chromatography,^5–7^ electrophoresis,^8,9^ and spectrofluorimetry.^10^ These methodologies generally demand laborious and lengthy sample preparation, including solvent-based extractions, excipient precipitation, and cleanup operations, which may reduce the reproducibility of results.^11^ In light of these limitations, electroanalytical techniques have become a promising alternative, delivering advantages such as ease of use and rapid analysis, high sensitivity, and reduced need for elaborate sample pretreatment besides the possibility of miniaturization of the system and in situ analysis. Some electroanalytical methods have been reported for QTP determination using carbon paste-modified electrodes,^12–14^ coated wire electrodes,^15^ modified glassy carbon electrodes,^16,17^ bare glassy carbon electrodes,^18–20^ and mercury electrodes.^21^ Electropolymers have recently been increasingly employed in analytical methodologies. Among these applications, the electropolymerization of amino acids on electrode surfaces has emerged as a promising strategy for determining pharmaceutical compounds in various matrices. This approach enables the formation of selective and functionalized films directly on the electrode, enhancing the performance of the electrochemical sensors.

The generation of homogeneous, stable, and chemically functional polymeric films is the benefit of electropolymerization of amino acids. These coatings yield selective recognition regions, promoting specific interactions with target analytes and consequently enhancing the analytical sensitivity and selectivity of the system.^22^ The polymerization process begins with electrochemical oxidation of the amino functional group, which generates reactive intermediates under an applied potential. These intermediates can form covalent bonds with neighboring monomer units, resulting in the formation of radical species that adhere strongly to the electrode surface. Additionally, the diverse functional groups present in the side chains of amino acids create distinct chemical microenvironments, which influence the properties of the resulting polymeric film.^23,24^ Certain amino acids have been used for this application, such as glycine,^25–27^ methionine,^28–30^ l-serine,^31,32^ and l-cysteine.^33–35^ However, to the best of our knowledge, the application of a poly­(l-cysteine)-modified glassy carbon electrode as an electrochemical sensor for QTP quantification has not been previously reported.

Therefore, the study aimed to develop an electroanalytical approach for the quantification of QTP in tablets by investigating its electrochemical interaction with a poly­(l-cysteine)-modified glassy carbon electrode (poly­(l-cys)/GCE). The method proved to be simple, selective, accurate, and rapid for the quantitative determination of QTP in commercial drug tablets without any previous sample preparation procedure. Owing to these characteristics, the proposed electroanalytical approach aligns with several Sustainable Development Goals (SDGs) of the 2030 Agenda, particularly SDG 6 (Clean Water and Sanitation), SDG 9 (Industry, Innovation, and Infrastructure), and SDG 14 (Life Below Water).

## 2 Experimental Section

### 2.1 Chemicals, Reagents, and Solutions

The QTP was purchased from Merck; the magnesium stearate from Êxodo Cientifica (Sumaré, Brazil); the starch, calcium phosphate, and talc from Synth (Diadema, Brazil); the povidone (Vetec) and potassium ferricyanide from Sigma-Aldrich (St. Louis, MO, USA); the potassium chloride and sodium hydroxide from Proqumios (Rio de Janeiro, Brazil); and the hydrochloric acid and acetic acid from Neon (Sao Paulo, Brazil). The pH of the acetate buffer solution (ABS) was adjusted to the desired values with 1.0 mol L^–1^ hydrochloric acid and 1.0 mol L^–1^ sodium hydroxide solutions. Stock QTP solutions with concentrations of 1.0 mmol L^–1^ were prepared in 0.10 mol L^–1^ ABS (pH 4.0). Diluted standard QTP solutions were prepared from the stock solution through appropriate dilution with the supporting electrolyte. All reagents were of analytical grade and utilized directly as supplied, without further treatment. All solutions were prepared using ultrapure water.

### 2.2 Apparatuses

Voltammetric and electrochemical impedance spectroscopy (EIS) tests were performed on an Autolab PGSTAT 128N potentiostat/galvanostat (Utrecht, Netherlands) using Autolab Nova, version 2.1.3, software for data collection and analysis. The experimental setup consisted of a three-electrode glass cell equipped with a glassy carbon electrode (GCE) (diameter of 5.0 mm) as the working electrode, Ag/AgCl (3.0 mol L^–1^ KCl) as the reference, and a 1.0 cm^2^ platinum wire as an auxiliary electrode, which was used for the electrochemical measurements.

The spectrophotometric measurements were carried out by using a Genesys 10 UV model (Thermo Scientific). The pH of all solutions was measured by using a model PS3-E (Ion) pH meter calibrated with standard buffer solutions. All measurements were carried out at room temperature.

### 2.3 Poly­(l-cys)/Electrode Sensor Fabrication

The GCE surface was first polished with an alumina (0.05 μm) slurry on a polishing pad until a mirror-like appearance was obtained. The electrode was then rinsed with ultrapure water and allowed to dry at room temperature. The electropolymerization of monomer l-cysteine on the GCE surface was carried out through successive cycles with cyclic voltammetry. The monomer concentration of l-cysteine was 5.0 mmol L^–1^ prepared in 0.1 mol L^–1^ KCl (pH 4.0). The GCE surface was completely electropolymerized through 20 cycles using an anodic scan in the potential range of −0.6 (initial potential) to +2.0 V (vertex potential) at 100 mV s^–1^ over to 1040 s. The poly­(l-cys)/GCE was thoroughly rinsed with ultrapure water for removing weakly adsorbed molecules and placed into the electrochemical cell.

### 2.4 Electrochemical Film Characterization: Cyclovoltammetric Studies and Electrochemical Impedance Spectroscopy

The characterization of the poly­(l-cys)/GCE was performed by using cyclic voltammetry (CV) using a PGSTAT 128N potentiostat/galvanostat (Metrohm Autolab B.V., Utrecht, The Netherlands) in a conventional three-electrode electrochemical cell. The modified glassy carbon electrode (poly­(l-cys)/GCE) was used as the working electrode, with a Ag/AgCl (KCl-saturated) electrode serving as the reference, and a platinum wire as the counter electrode. CV experiments were carried out in 0.1 mol L^–^^1^ KCl solution, within a potential window from −0.1 to 1.3 V (vs Ag/AgCl), while varying the scan rate from 10 to 220 mV s^–1^. Current, potential, and time data were recorded and processed to determine the voltammetric charge (*q*) by integrating the anodic and cathodic branches of the voltammograms, enabling the evaluation of the redox reversibility. In addition, the differential capacitance (*C*_d_), associated with the electrical double layer at the solid–liquid interface, was estimated based on the linear correlation between capacitive current (*i*_c_) and scan rate (*v*), according to the adaptation by Da Silva et al.^36^

In an electrochemical impedance spectroscopy study, measurements were conducted at the formal potential of the [Fe­(CN)_6_]^3–^/[Fe­(CN)_6_]^4^^–^ redox couple (1.0 mmol L^–^^1^, 1:1 ratio), in the presence of a supporting electrolyte containing 0.10 mol L^–1^ KCl. Data fitting was performed using a software tool implementing the simplex optimization algorithm combined with nonlinear least-squares fitting procedures. Impedance spectra were recorded over a frequency range spanning from 0.1 to 1 × 10^5^ Hz. The charge-transfer resistance (*R*_ct_) values were extracted by applying nonlinear regression analysis to the semicircular region of the Nyquist plots (*Z*_Re_ vs *Z*_i__m_).

### 2.5 Electrochemical Measurements

First, QTP solutions (1.0 mmol L^–1^) were prepared by dissolving the required amount of the stock solutions in 0.10 mol L^–1^ ABS (pH = 4.0). A volume of 10.0 mL of each solution was then placed in the electrochemical cell, and the cyclic voltammogram was recorded at 100 mV s^–1^ in the potential range from −0.1 to +1.4 V using bare and modified glassy carbon electrode surfaces (electrochemical behavior). The effect of pH on the determination of QTP was also investigated using CV at 100 mV s^–1^ in 0.10 mol L^–1^ ABS over the pH range of 2.0–10.0. The relationship of peak potential, peak current, and pH values was plotted in a graph, and the pH value choices were based on the best analytical signal. The pH value was constant for further analysis. In scan rate studies, a volume of 10.0 mL of the QTP solutions (1.0 mmol L^–1^) and 0.10 mol L^–1^ ABS (pH= 4.0) was placed in an electrochemical cell and the scan rate was varied over the range of 10–300 mV s^–1^. Cyclic voltammograms were recorded, and *I*_p_ vs *v* ^0.5^ and log *I*_p_ vs log *v* plots were obtained to characterize the mass transfer mechanism. Square wave voltammetry (SWV) was employed to develop the analytical method, with optimization of the parameters (pulse amplitude, step increment, and frequency) to ensure optimal performance for the quantification of QTP in pharmaceuticals. To achieve optimal experimental conditions, the potential pulse amplitude was systematically varied from 10 to 100 mV, while maintaining the potential step increment and frequency constant at 5 mV and 40 s^–^^1^, respectively. Subsequently, the influence of the potential step increment was investigated within the range 1–10 mV, with the potential pulse amplitude and frequency held constant at 60 mV and 40 s^–^^1^, respectively. Lastly, the frequency was varied between 10 and 100 s^–^^1^, while fixing the potential pulse amplitude and potential step increment at 60 and 5 mV, respectively. As a result of these optimizations, the square wave voltammetric parameters were established as follows: a potential pulse amplitude of 60 mV, a potential step increment of 5 mV, and a frequency of 50 s^–^^1^. After optimized conditions (pH, electrolyte support, and SWV parameters), calibration curves, detection and quantification limits, and intraday and interday precision were calculated according to IUPAC recommendations, and the QTP concentration in the tablets was determined by the standard addition method. Interference studies were performed by evaluating the analytical signal of QTP in the presence of substances present in pharmaceutical tablets at a ratio of 1:50.

### 2.6 QTP Analysis on Pharmaceutical Tablets

The proposed poly­(l-cys)/GCE sensor was used to investigate real samples through direct analysis of QTP in pharmaceutical formulations (tablets), for which QTP tablets were acquired from a local drugstore. Based on the manufacturer’s specifications, each tablet contained 200 mg of QTP. Ten tablets were ground to a powder, and approximately 200 mg of QTP was accurately weighed, dissolved in ultrapure water with 10 min of magnetic stirring, and transferred to a 100.0 mL volumetric flask, then brought to volume using 0.1 mol L^–^^1^ ABS buffer at pH 4.0. It was subsequently diluted with 0.1 mol L^–1^ ABS (pH 4.0) to the final volume of the volumetric flask. This solution was filtered, and an aliquot of 100 μL was transferred to a 10 mL volumetric flask and diluted with a supporting electrolyte to the final volume (test solution). For analysis, the solution was placed in an electrochemical cell, and the concentration of QTP was measured employing the standard addition method. The results obtained were compared with an official United States Pharmacopeia protocol.

## 3 Results and Discussion

### 3.1 Electropolymerization of l-Cysteine on a Glassy Carbon Electrode Surface

Amino acid polymeric films have been applied to electrodes to increase the electron transfer kinetics and charge transfer. In these cases, the number of cycles can be used to control the thickness of the film, which affects the stability and repeatability of the sensor. The electropolymerization of l-cysteine in 5.0 mmol L^–1^ l-cysteine was carried out in the range of −0.6 to 2.0 V (vs Ag/AgCl) in the current investigation. During the electropolymerization process, the film thickness was measured from the reverse scan peak reduction at +0.7 V (Figure 1, zoomed image). This peak reduction can be attributed to the reduction of the −SH group from the l-cysteine residues. The thickness of the polymeric film was evaluated by monitoring of the reduction peak at +0.7 V at different cycles. The relationship of the number of cycles of the electropolymerization procedure and reduction current is shown in Figure S2. After the 20th cycle, the peak remained constant, indicating the end of the procedure (Figure 1). The influence of thickness of the polymeric film on QTP oxidation was also evaluated. At different electrodes with a variable number of cycles on the electropolymerization procedure, the oxidation peak of QTP was recorded and it was observed that after the 20th cycle, the oxidation current peak was constant. The results are shown in Figure S3.

**Figure 1:**
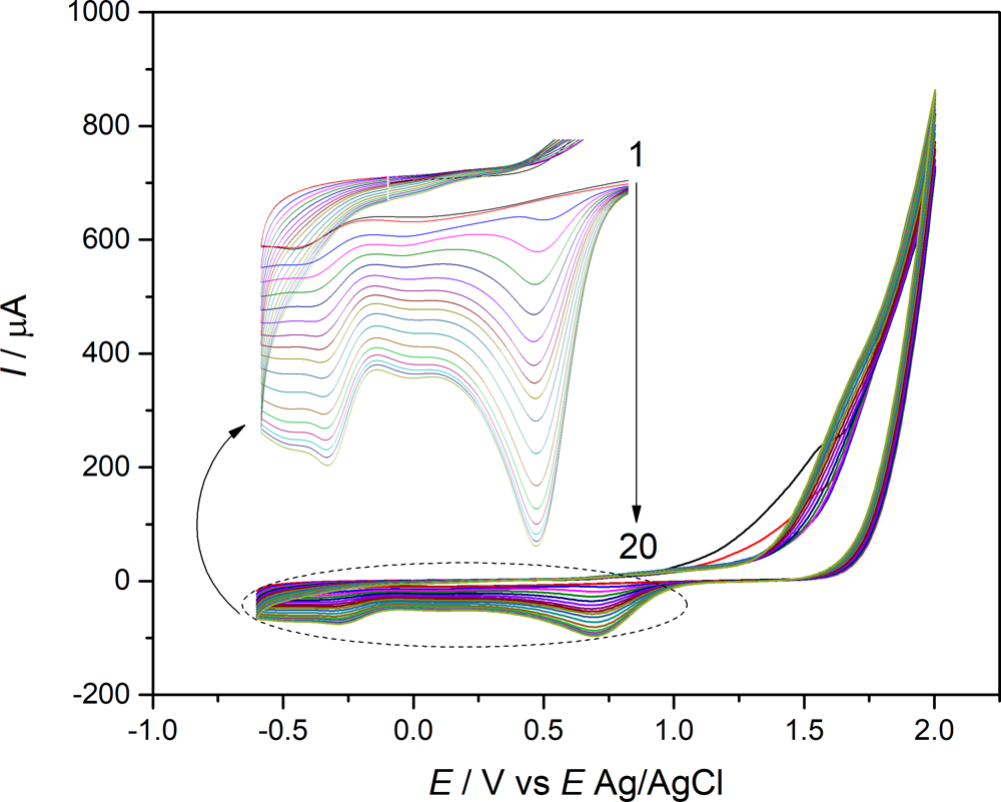
Cyclic voltammograms of l-cysteine electropolymerization on the GCE. *C*_l‑cysteine_ = 5.0 mmol L^–1^ in 0.10 mol L^–1^ KCl solution, pH = 4.0, and υ = 100 mV s ^–1^.

The electropolymerization of poly­(l-cysteine) (poly­(l-cys)) onto a glassy carbon electrode is initiated by the oxidative activation of the amino group, which occurs around +1.6 V during the first anodic scan in cyclic voltammetry. This peak typically diminishes in subsequent scans, indicating progressive surface passivation due to polymer growth. As illustrated in Figure 2, the polymerization mechanism proceeds through multiple steps: initially, the amino group undergoes oxidation to generate a radical cation. Subsequently, the l-cysteine molecule forms a covalent C–N bond with the electrode surface accompanied by the loss of one electron and one proton, anchoring the monomer. In an acidic medium, the carboxyl group becomes protonated, enhancing its electrophilic character and facilitating a nucleophilic attack by a second l-cysteine molecule. This interaction leads to intramolecular proton transfer, resulting in water elimination and subsequent deprotonation of the carbonyl group. The overall process culminates in the formation of amide linkages, promoting the propagation of a linear polymeric structure on the electrode surface.

**Figure 2:**
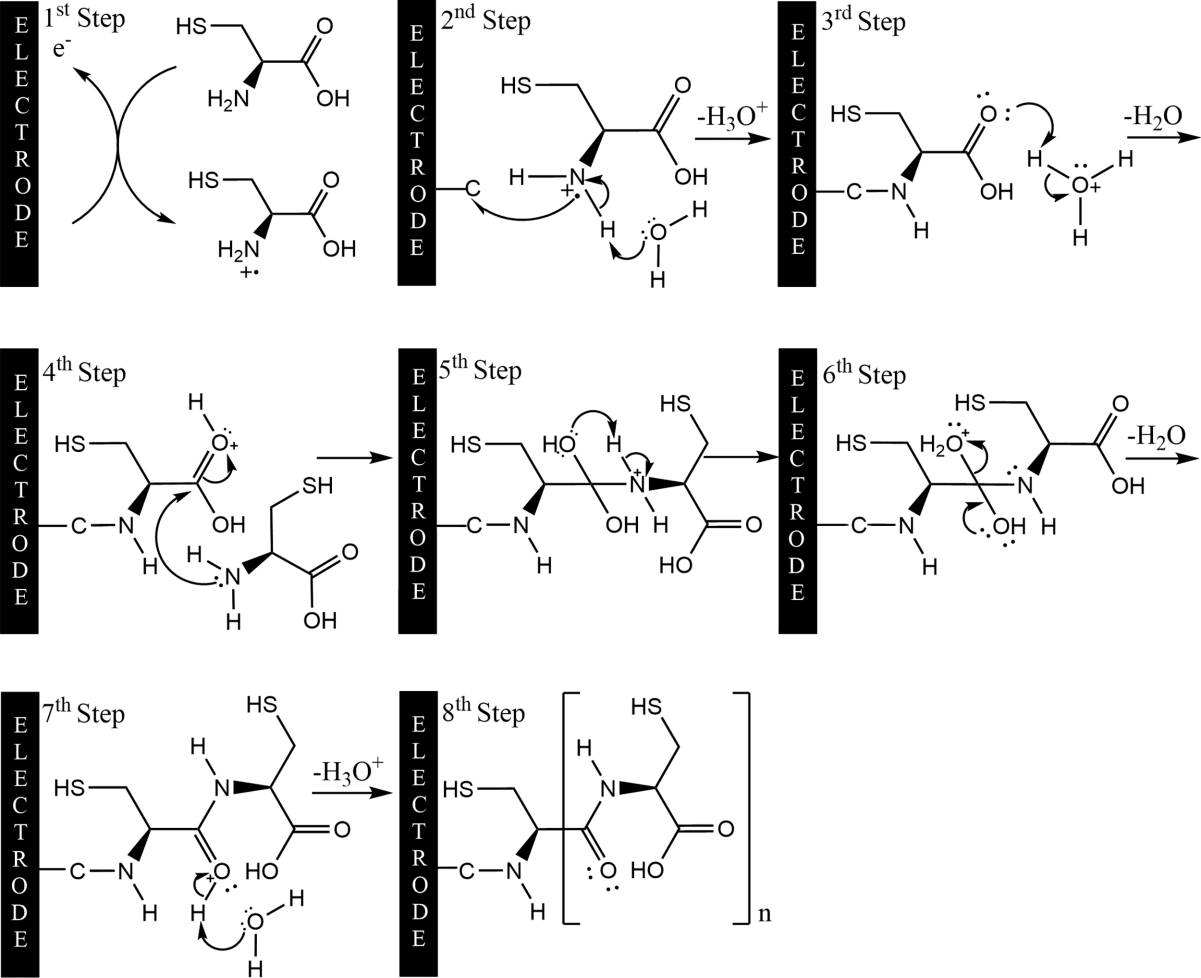
Proposed mechanism of the electropolymerization of l-cysteine at GCE.

### 3.2 Electrochemical Impedance Spectroscopy (EIS) Analysis

Electrochemical impedance spectroscopy (EIS) is a widely employed technique in the development of electrochemical sensors due to its high sensitivity, precision, and ability to provide detailed information about interfacial processes occurring at the electrode surface. The resulting data are typically displayed as Nyquist plots, which exhibit two distinct regions: a semicircular arc at high frequencies, corresponding to charge-transfer resistance, and a linear region at low frequencies, associated with diffusion-controlled processes. The diameter of the semicircle provides a direct estimate of the charge-transfer resistance (*R*_ct_), reflecting the kinetics of the redox process at the electrode/solution interface.^27^ Figure 3 presents the impedance spectra in the form of Nyquist plots, highlighting both the real (*Z*_Re_) and imaginary (*Z*_i__m_) components of impedance for the bare glassy carbon electrode (GCE) and the electrode modified with poly­(l-cysteine) (poly­(l-cys)/GCE).

**Figure 3:**
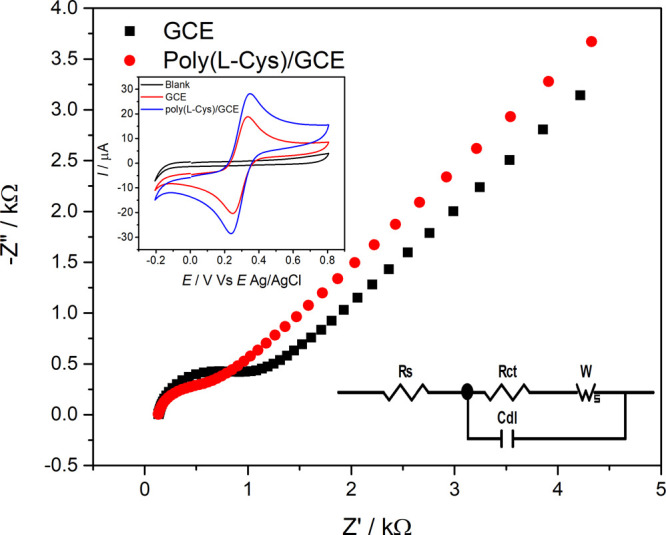
Nyquist plot of 1.0 mmol L^–1^ [Fe­(CN_6_]^3–^/Fe­(CN_6_]^4–^ solution in 0.10 mol L^–1^ KCl for bare GCE and poly­(l-cys)/GCE. Working electrode potential: +0.2 V. Inset: Cyclic voltammograms of 1.0 mmol L^–1^ [Fe­(CN_6_]^3–^/Fe­(CN_6_]^4–^ on different electrodes. Selected regions in the Nyquist plot and standard Randles equivalent-model circuit were applied for calculations and the cyclic voltammograms of the [Fe­(CN_6_]^3–^/Fe­(CN_6_]^4–^ system.

The analysis of the data obtained by EIS was done by recording the experimental data, and an equivalent circuit model based on the modified Randles circuit was obtained (illustrated in the inset of Figure 3). This model consists of the solution resistance (*R*_s_) in series with a constant phase element (CPE), which represents the double-layer capacitance (DLC). The CPE is arranged in parallel with the charge-transfer resistance (*R*_ct_), which is further connected in series with the Warburg impedance (*W*), accounting for the diffusional transport of the ferri-ferrocyanide redox species from the bulk solution to the electrode surface.

In the high-frequency region, the appearance of a semicircular arc in the Nyquist plot corresponds to a charge-transfer-limited process. The diameter of this semicircle is directly proportional to the charge-transfer resistance (*R*_ct_). The extracted values of *R*_s_, *R*_ct_, DLC, and *W* for each electrode configuration are summarized in Table 1.

**Table 1:** Nyquist Plot Parameters Obtained for Bare GCE and Poly­(l-cys)/GCE

electrodes	*R*_s_ (Ω)	*R*_ct_ (Ω)	*W* (μmho)	DLC (μF)
bare GCE	140.0	726.0	268.0	1.85
poly(l-cys)/GCE	144.0	486.0	405.0	9.75

A semicircle was observed for the unmodified glassy carbon electrode (GCE), with an *R*_ct_ of 726 Ω, indicating limited interfacial electron transfer. The *R*_ct_ is a parameter that is related to that kinetic reaction; thus, the rate of the reaction under study is inversely proportional to the resistance to charge transfer on the electrochemical system. Upon modification with poly­(l-cysteine), the charge-transfer resistance decreased to 486 Ω for the poly­(l-cys)/GCE system. This result demonstrates a significant increase in electron transfer kinetics, reflecting an increase in the kinetics of the ferricyanide/ferrocyanide redox reaction. This effect may be due to the phenomenon of hyperconjugation of orbitals between the bonds in the l-cysteine molecules adsorbed on the surface of a GCE.

### 3.3 Voltammetric Charges, Differential Capacitance, and Roughness Factor of the Poly­(l-cysteine) Film

Cyclic voltammetry was employed to evaluate the electrochemical behavior of the poly­(l-cysteine) (poly­(l-cys)) film, aiming to quantify both anodic and cathodic charges and their respective ratios. This voltammetric analysis offers valuable insights into the physicochemical characteristics of the material’s surface, whether homogeneous or presenting roughness, applicable to solid substrates and polymeric coatings alike. Through this technique, it is possible to discern the presence and types of redox phenomena or, alternatively, processes associated with electric double-layer charging.^37^ The quantitative data obtained, including the anodic and cathodic charges and their ratios, are summarized in Table 2.

**Table 2:** Voltammetric Charges and Their Ratios Obtained from CV Experiments Using Poly­(l-cys)/GCE in 0.1 mol L^–1^ KCl Solution^a^

electrode	*N* (mV s^–1^)	qa (C)	qc (C)	|qa/qc|
poly(l-cys)/GCE	10	1.611 × 10^–4^	–1.650 × 10^–5^	9.76
	30	9.550 × 10^–5^	–1.433 × 10^–5^	6.67
	50	1.015 × 10^–4^	–1.885 × 10^–5^	5.38
	70	8.890 × 10^–5^	–1.966 × 10^–5^	4.52
	90	8.798 × 10^–5^	–1.596 × 10^–5^	5.51
	120	6.350 × 10^–5^	–1.453 × 10^–5^	4.37
	150	7.112 × 10^–5^	–1.814 × 10^–5^	3.92
	180	7.167 × 10^–5^	–2.040 × 10^–5^	3.51
	220	7.988 × 10^–5^	–2.023 × 10^–5^	3.95

a Experimental conditions = supporting electrolyte: KCl, 0.1 mol L^–1^; Δ*E* = 0.1–0.5 V vs Ag/AgCl; Δν: 10–220 mV s^–1^.

The data reveal that the poly­(l-cysteine) (poly­(l-cys)) film exhibited voltammetric charges of similar magnitude, particularly at lower scan rates. Charges in the microcoulomb (μC) range suggest that charge-transfer processes are significantly limited. This behavior can be attributed to one or more of the following factors: (i) a low density of redox-active sites on the electrode surface, (ii) the formation of ultrathin polymeric films, or (iii) the predominance of capacitive over Faradaic processes.^38,39^ In the case of electrogenerated polymer films like poly­(l-cysteine), such low charge values may also reflect a limited degree of polymerization or the essentially nonredox nature of the film within the studied potential window.^40^

Moreover, the low voltammetric charges allow inferences regarding charge migration within the polymer matrix. In the present study, the obtained results suggest that the film possesses a smooth and compact morphology, with the occurrence of electronic processes predominantly on the external film’s surface.^41,42^

The ratio between anodic and cathodic charges also offers valuable insight into the reversibility of the electrochemical processes involved. This is particularly relevant when considering mechanisms such as the chemisorption of electroactive species onto the functional groups of poly­(l-cysteine) or reversible structural/electronic rearrangements within the film itself during potential cycling. Notably, the observed |qa/qc| ratios consistently exceeded 1.0 across all scan rates tested, showing a variation of 59.52% between 10 and 220 mV s^–1^.^34,43^

Charge ratios greater than unity (|qa/qc| > 1.0) are typically indicative of irreversible redox behavior, where the charging and discharging of the electrical double layer occur asymmetrically, deviating from the behavior expected in ideal capacitive systems. In this context, electronic-level changes within the film’s functional groups likely play a crucial role in shaping the voltammetric response observed experimentally.^44,45^

In addition to the analysis of voltammetric charges, a complementary approach involves evaluating the differential capacitance and the intrinsic roughness factor of the poly­(l-cysteine) film. Typically, a linear relationship is observed between the capacitive current (*i*_c_), measured at a fixed potential, and the scan rate. From this relationship, the differential capacitance (*C*_d_) is determined as the slope of the linear fit, providing valuable information about changes in surface properties, including morphological features and the extent of film coverage on the electrode.^34,36^

Variations in the surface charge distribution as a function of the scan rate may be influenced by spatial inhomogeneities in the electric field across the electrode or the polymeric film. These nonuniformities often arise from differences in film thickness, local conductivity, or structural irregularities, all of which can significantly impact the observed capacitive response.^46^

The interpolation of capacitive current data versus scan rate revealed two distinct linear regimes (Figure S1). The first, observed at lower scan rates, corresponds to the total capacitance of the film, encompassing contributions from both the surface and bulk of the material. The second, which emerges at higher scan rates, reflects the capacitive response primarily associated with the outermost regions of the film, where charge redistribution occurs more rapidly. According to Da Silva et al.,^36^ the roughness factor (φ) can be estimated by the ratio between the inner and total capacitances, offering a metric for assessing the degree of surface heterogeneity. Figure 4 presents the cyclic voltammetry profiles obtained in 0.1 mol L^–1^ KCl solution, used for these determinations.

**Figure 4:**
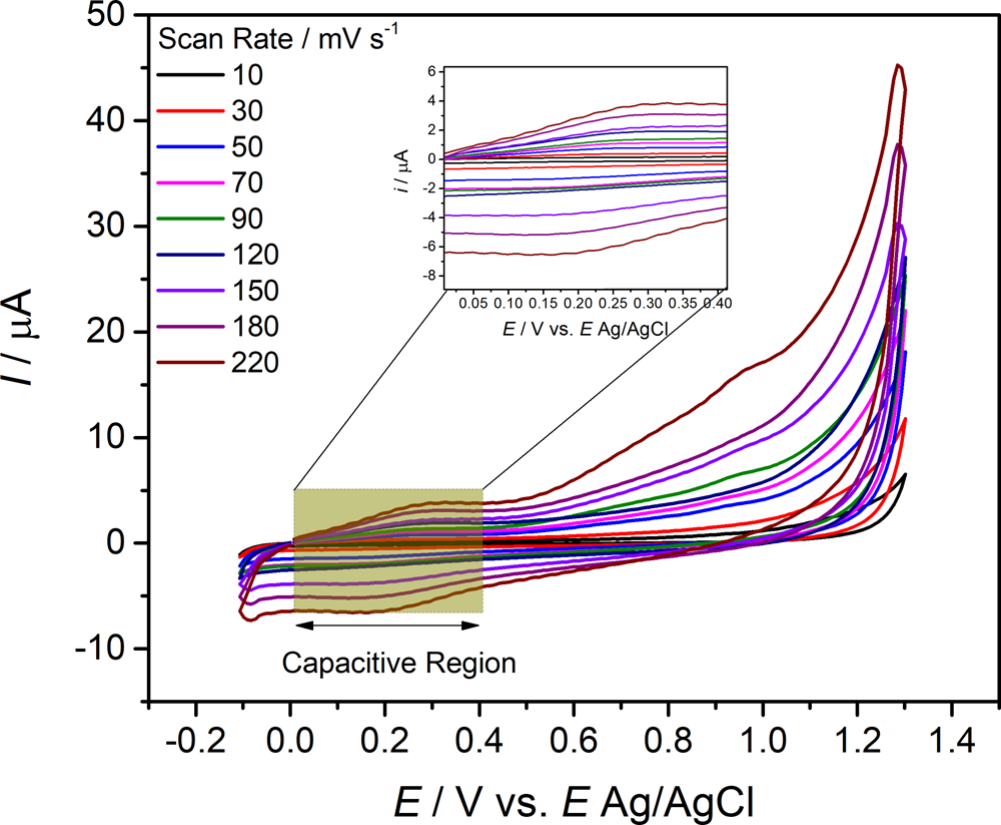
CV profiles recorded at different scan rates, highlighting the capacitive region used to calculate differential capacitances and the film roughness factor. Experimental conditions: potential window of 0.1–0.5 V vs Ag/AgCl; KCl concentration = 0.1 mol L^–1^; scan rate range = 10–220 mV s^–1^.

Table 3 presents the differential capacitance values of the poly­(l-cysteine) film, along with the corresponding roughness factor. These parameters were derived from the linear regression domains observed in the capacitive current versus scan rate plots, as outlined in eqs 1, 2, and 3 and illustrated in Figure S1.

**Table 3:** Differential Capacitances and Roughness Factors Calculated for the Poly­(l-cys)/GCE Electrode in 0.1 mol L^–1^ KCl Solution, within the Potential Range Corresponding to the Capacitive Current Region

electrode	*E*_c_ (V vs Ag/AgCl)	*C*_d_ (μF)	*C*_d,e_ (μF)	*C*_d,I_ (μF)	φ
poly(l-cys)/GCE^a^	0.1	6.77	6.66	0.11	0.016
	0.2	12.97	12.63	0.34	0.026
	0.3	15.89	14.44	1.45	0.091
	0.4	16.55	16.19	0.36	0.022
	0.5	19.89	18.88	1.01	0.051

a Experimental conditions = supporting electrolyte: KCl, 0.1 mol L^–1^; Δ*E* = 0.1–0.5 V vs Ag/AgCl; Δν: 10–220 mV s^–1^.

As shown in Table 3, the differential capacitance values remained below 20 μF, with a predominant contribution from the external capacitive component relative to the total differential capacitance. This indicates that the external surface of the film plays a more significant role than the internal regions in charge storage and accessibility, particularly during the charging of the electric double layer.

With respect to the roughness factor, the calculated φ values were consistently lower than 0.1000, which points to a film with a relatively compact and uniform morphology.^47,48^ Taken together, the low roughness factor and the differential capacitance results support the conclusion that the poly­(l-cysteine) film formed under the studied conditions is compact, smooth, and structurally homogeneous.

### 3.4 Electrochemical Behavior of QTP on the Bare GCE and Poly­(l-cysteine)-Modified Electrode

The electrochemical behavior of 1.0 mmol L^–1^ QTP in 0.10 mol L^–1^ KCl (pH = 4.0) for the different electrodes was investigated using CV at 100 mV s^–1^ over the potential range from −0.1 to +1.6 V vs Ag/AgCl. A poor irreversible oxidation peak referring to QTP at +1.25 V (Figure 5, line b) was obtained on the bare GCE. Under the same experimental conditions, on poly­(l-cys)/GCE, the QTP showed a well-defined irreversible peak at +1.15 V (Figure 5, line d). The lines (a) and (c) refer to the blank. The potential shift of 100 mV close to zero indicates enhanced the electrocatalytic properties of the immobilized film and a faster electron transfer reaction on the electrode surface.

**Figure 5:**
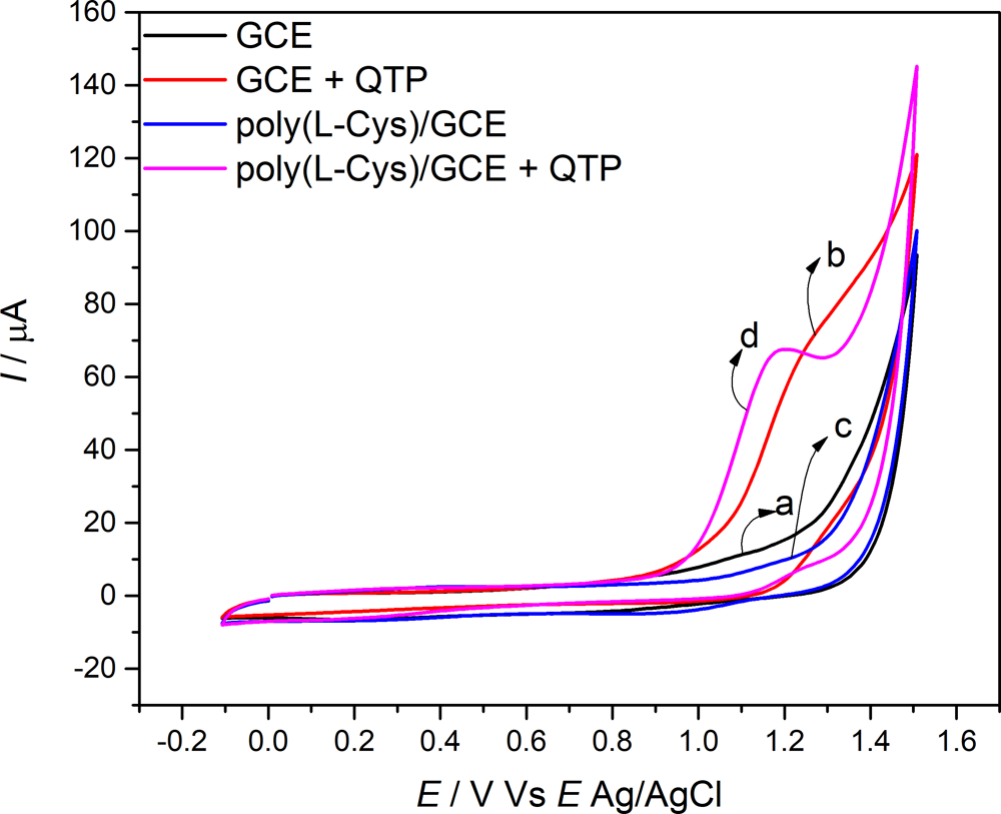
Cyclic voltammograms on GCE in (a) the absence and (b) the presence of 1.0 mmol L^–1^ QTP and cyclic voltammograms on poly­(l-cys)/GCE in (c) the absence and (d) the presence of 1.0 mmol L^–1^ QTP. *v* = 100 mV s ^–1^, electrolyte: 0.10 mol L^–1^ KCl (pH = 4.0).

### 3.5 Investigation of the Scan Rate Effects in Cyclic Voltammetry

The scan rate (*v*) was investigated using CV in the range of 10–300 mV s^–1^, as shown in Figure 6. The anodic peak currents of QTP, 1.0 mmol L^–1^ in 0.10 mol L^–1^ KCl solution (pH = 7.0), exhibited a positive correlation with the scan rate but was not linearly proportional (*R* = 0.97) to the square root of the scan rate within the studied range, as shown in Figure 6 (inset A). This indicates that the electrode reactions of QTP are not controlled by diffusion on the poly­(l-cys)/GCE surface. Linear regression equations between *I*_p_ and *v* ^0.5^ can be expressed by eq 1

$$I\;(A)=-1.2853\times 10^{-5}(\pm 1.86\times 10^{-6})+3.9\times 10^{-6}(\pm 1.73\times 10^{-6})v^{0.5}\;(\mathrm{mV}\;s^{-1})\;(R^{2}=0.97)$$ 1

**Figure 6:**
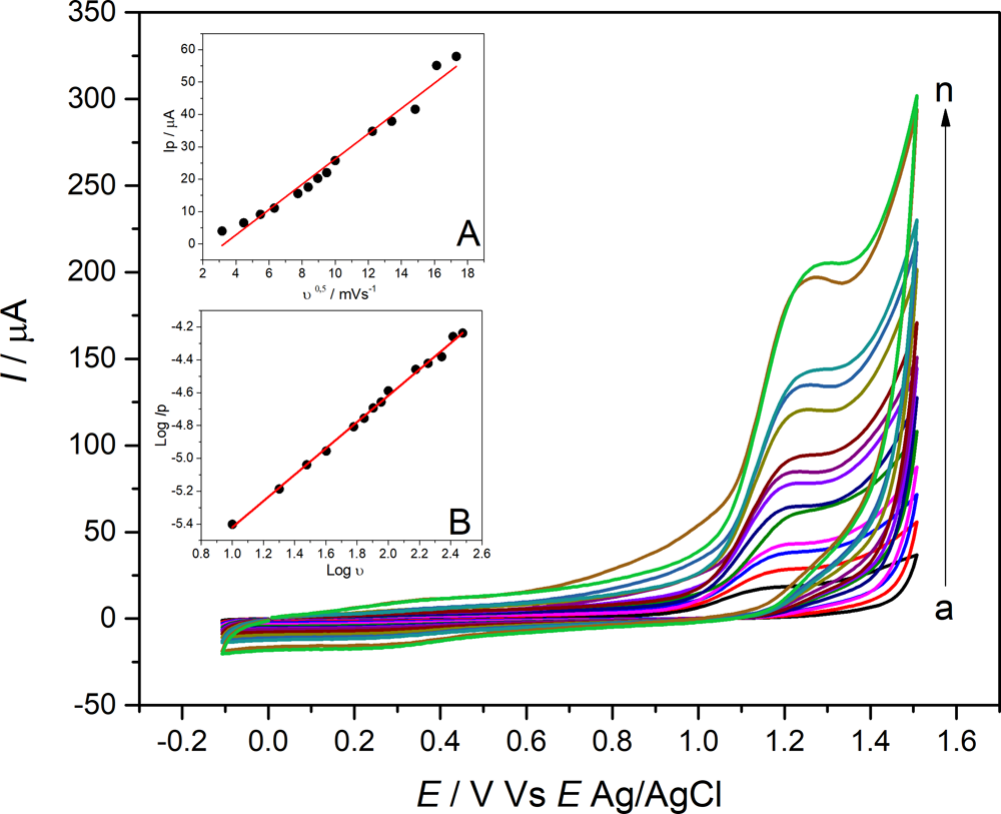
Cyclic voltammograms of 1.0 mmol L^–1^ QTP in 0.10 mol L^–1^ KCl (pH = 7.0) recorded with poly­(l-cys)/GCE at different scan rates: (a) 10, (b) 20, (c) 30, (d) 40, (e) 60, (f) 70, (g) 80, (h) 90, (i) 100, (j) 150, (k) 180, (l) 220, (m) 260, and (n) 300 mV s^–1^. Inset: (A) *I*_p_ vs *v* ^0.5^ plot. (B) log *I*_p_ vs log *v* plot.

In addition, the relationship between log *I*_p_ and log *v* is shown in Figure 6 (inset B). In this figure, a linear correlation between log *I*_p_ and log *v* can be observed and is expressed by eq 2.

$$\log I_{p}(A)=-6.22(\pm 0.02)+0.8(\pm 0.01)\;\log v\;(R^{2}=0.997)$$ 2

The slope of the log *I*_p_ vs log *v* plot was equal to 0.8. This result indicates that the QTP oxidation process on the electrochemical sensor is governed by a mixed mechanism (diffusion and adsorption), predominantly adsorption-controlled.^49^

### 3.6 Effect of pH on QTP Oxidation

In aqueous solution, the transfer of protons from or for organic molecules is generally fast and there is an equilibrium of protons next to the electrode surface, justifying this investigation. The role of pH in QTP oxidation was examined via CV at 100 mV s^–^^1^ in 0.10 mol L^–^^1^ ABS, with the supporting electrolyte pH spanning from 2.0 to 10. As illustrated in Figure 7, the peak potential and current were dependent on pH, indicating proton involvement in the oxidation.

**Figure 7:**
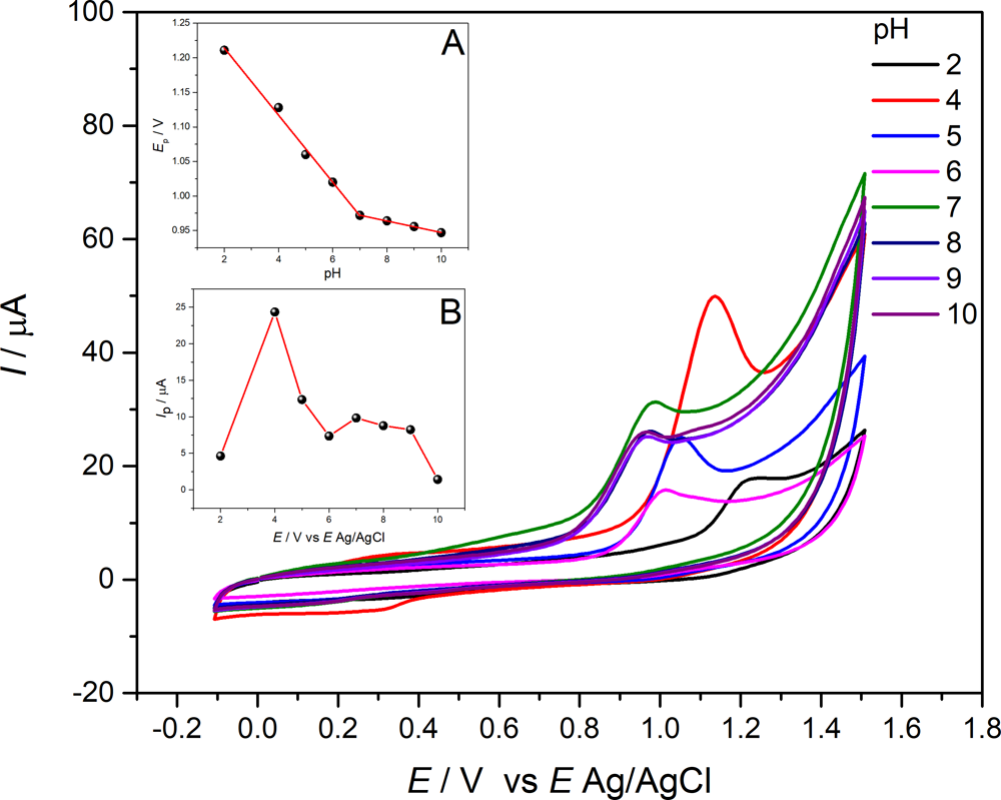
Cyclic voltammograms of 1.0 mmol L^–1^ QTP in 0.10 mol L^–1^ acetate buffer at different pH values obtained with poly­(l-cys)/GCE: *v* = 100 mV s^–1^. Inset A: (*E*_p_) vs pH plot; inset B: *I*_p_ (μA) vs *E* (V) relationship.

QTP peak currents exhibited an increase with a pH up to 4.0, followed by a decrease at higher pH levels. Therefore, pH 4.0 was adopted for subsequent experiments due to its optimal peak current. The peak potential shifted toward more negative potentials as the pH increased (eq 3). In the investigated pH range, two regions of linearity were observed. The intercept of these lines provides the p*K*_a_ value of the molecule in the current study.^50^ The value found was 6.8, which is close to that reported in the literature of 6.97 for QTP.^51^ The relationship between peak potential (*E*_p_) and supporting electrolyte pH, in addition to *I*_p_ vs *E*, is shown as insets in Figure 7.

$$E_{p}\;(V)=1.31(\pm 0.01)-0.050(\pm 0.002)\mathrm{pH}\;(R^{2}=0.994)$$ 3

The slope was estimated as −50 mV at pH^–1^. Negative slopes can be associated with the deprotonation reaction in the oxidation process, which is facilitated at high pH values.^52^ The slope is close to the theoretical value of the Nernst equation, suggesting that the number of protons that participated in the QTP oxidation reaction was equal to the number of transferred electrons and the QTP oxidation is pH-dependent.^53^ The number of electrons was experimentally calculated by using eq 4 for the irreversible process.

$$E_{p}-E_{p}/2=\frac{47.7\;\mathrm{mV}}{\alpha \times n}$$ 4

where *E*_p_ is peak potential, *E*_p_/2 is peak potential half height, α is the electronic transfer coefficient, and *n* is the number of electrons involved in QTP oxidation.

Considering that for irreversible processes, α can be approximated to 0.5,^39^ the number of electrons involved in QTP oxidation is close to one.

The peak potential of QTP at pH higher than about 7.0 remains practically constant. For pH < 7.0, the peak potentials shifted to less positive values as the pH increased (Figure 7A), suggesting an acid–base equilibrium with p*K*_a_ ≈ 7.0 in the electroactive region. When the pH exceeds p*K*_a_, the conjugate base dominates; at pH < p*K*_a_, it is generated through fast deprotonation of QTP. The p*K*_a_ value of QTP is around 6.8, and the piperazine moiety is almost completely protonated on both nitrogen atoms in buffer solution at pH 4.0.^51^ Ozkan et al.^18^ investigated other substances with a piperazine group in their structure, and the authors observed that when the aliphatic nitrogen of the piperazine rings is protonated (pH < p*K*_a_), an oxidation reaction occurs on the proximal nitrogen. These results suggest that the piperazine group is responsible for the oxidation reaction of QTP in ABS pH 4.0. These results suggest that the piperazine group is responsible for the oxidation reaction of QTP in ABS (pH 4.0).^18^ The reaction of QTP oxidation is shown in Figure 8.

**Figure 8:**
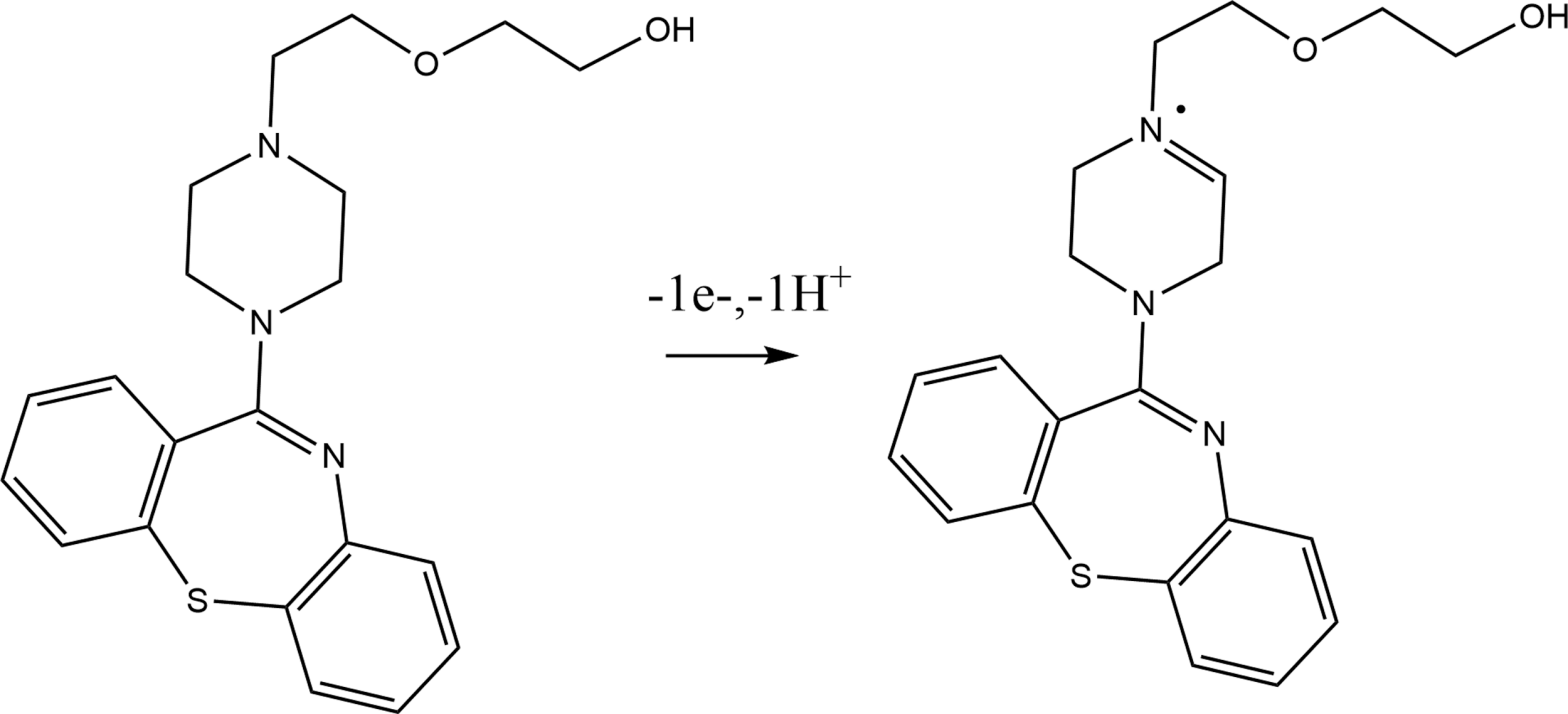
Electrochemical oxidation reaction of QTP on the poly­(l-cys)/GCE surface.

### 3.7 Electroanalytical Determination of QTP by Square Wave Voltammetry

The analytical determination of QTP square wave voltammetry (SWV) was used due to higher sensitivity, better signal resolution, and the lowest detection limit. The pulse amplitude (*a*), frequency (*f),* and step potential (Δ*E*_s_) parameters were optimized, and Figure 9 shows voltammograms for different QTP concentrations in 0.10 mol L^–1^ ABS (pH = 4.0) with applied potentials of 0.9–1.5 V, under optimized conditions (*a* = 60 mV, *f* = 50 s^–1^, and Δ*E*_s_ = 5 mV). Under optimized conditions, the QTP concentration varied over the range of 8.05–85.00 μmol L^–1^. The oxidation peak currents were linearly proportional to the QTP concentrations in the studied range. The relationship between current and QTP concentration can be expressed according to eq 5.

$$I_{p}\;(\mu A)=-0.33(\pm 0.2)+0.164(\pm 0.004)C_{\mathrm{QTP}}/\mu \mathrm{mol}\;L^{-1\;}(R^{2}=0.993)$$ 5

**Figure 9:**
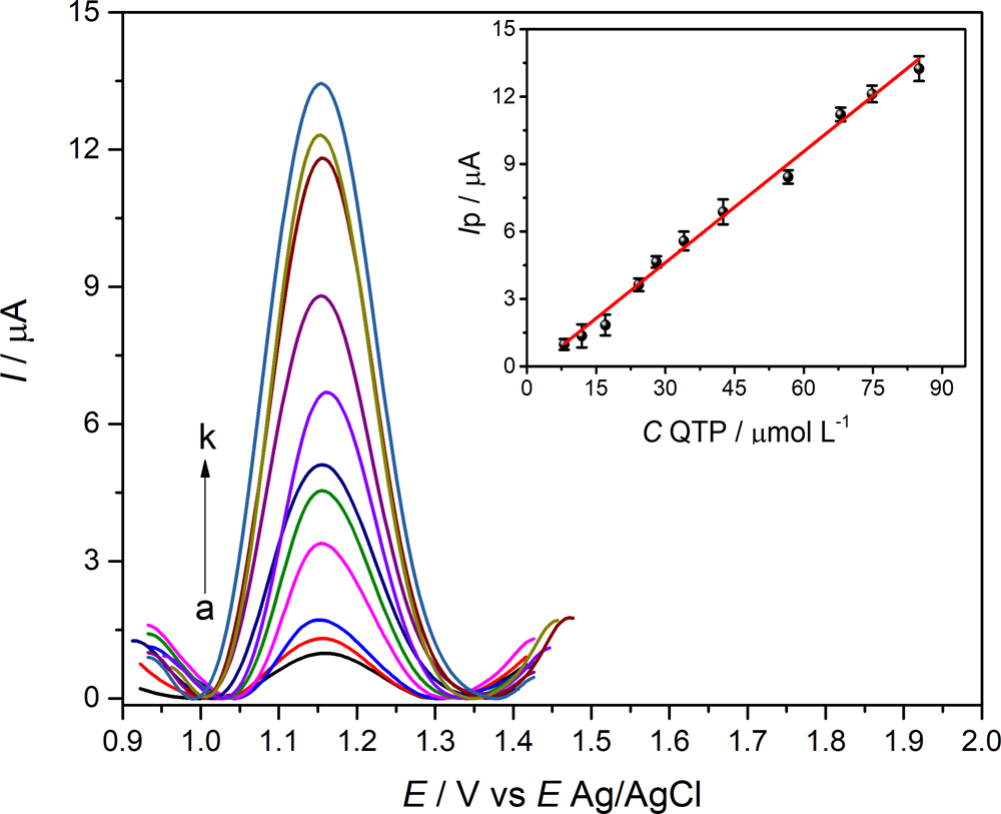
Square wave voltammograms of QTP solutions. (a) 8.05, (b) 11.90, (c) 17.10, (d) 24.22, (e) 28.05, (f) 34.20, (g) 42.50, (h) 56.61, (i) 68.00, (j) 74.80, and (k) 85.00 μmol L^–1^. Optimized SWV conditions: *a* = 60 mV, *f* = 50 s^–1^, and Δ*E*_s_ = 5.0 mV in 0.10 mol L^–1^ ABS (pH = 4.0) for poly­(l-cys)/GCE. Inset: *I*_p_ vs *C*_QTP_.

The limits of detection (LOD) and limits of quantification (LOQ) were calculated according to IUPAC^54^ (LOD = 3*s*_B_/*S* and LOQ = 10*s*_B_/*S* in which *s*_B_ is the standard deviation of the baseline noise and *S* is the slope of the calibration curve), considering 1.17 and 3.91 μmol L^–1^, respectively. The linear range was wide, and the limits of detection were low, like the LOD and LOQ values already reported for QTP through electrochemical methods, as compared in Table 4.

**Table 4:** Comparative Study between the Proposed Method and Recent Electrochemical Procedures for QTP Determination

method	electrode	pH	peak potential	linear range^a^	LOD^a^	sample
^9^SWV	Pd–Ag/g-C_3_N_4_/CPE	7.0	+0.9 V	5.26–107.14	1.5	tablet and human plasma
^10^DPV	CCN/LFMO/IL/CPE	7.0	+0.9 V	0.090–900	0.011	blood serum, urine, and tablets
^11^SWV	MIP-CPE	5.5	+1.2 V	0.016–2.5	0.00504	tablet and urine
^12^potentiometry	coated wire electrode	6.0		20–10,000	3.2	tablet and urine
^13^SWV	SBAP/GCE	3.0	+0.9 V	0.08–7.5	0.019	tablets, human serum, and urine
^14^DPAdSV	GnPs-Naf/GCE	7.0	+0.9 V	0.01–10	0.022	tablets and human urine
^15^OSWV	GCE	3.5	+1.0 V	4–200	0.133	tablets, human serum, and urine
^16^DPV	GCE	2.0	+0.65 V	0.02–5	0.01	tablets, human serum, and urine
SWV	poly(l-cys)/GCE	4.0	+1.1 V	8.05–85.0	1.17	tablets

a Concentration units: μmol L^–1^.

### 3.8 Intraday and Interday Precision

The intraday and interday precisions of the QTP peak currents for 34.20 μmol L^–1^ in 0.10 mol L^–1^ ABS (pH = 4.0) were evaluated through seven successive measurements of the peak current in the same solution and seven measurements of the peak current over 7 days, respectively. The RSD values obtained were 2.56 and 4.25% for QTP, respectively, suggesting adequate intraday and interday precision.

### 3.9 Study of Interference

The effect of common excipients in pharmaceutical formulations such as magnesium stearate, starch, povidone, talc, and calcium phosphate was investigated. Solutions of these compounds were freshly prepared in 34.20 μmol L^–1^ in 0.1 mol L^–1^ ABS (pH 4.0) of QTP solution with an interferent compound concentration ratio of 1:50. The analytical signal was monitored and compared with the signal obtained for the pure QTP solution (Table 5). The results revealed that these compounds did not interfere significantly with the sensor response, meaning that the proposed sensor exhibits good selectivity for QTP determination in pharmaceuticals tablets.

**Table 5:** Assessment of the Interference from Other Compounds during QTP Determination

interfering compound	relative response (%)
magnesium stearate	94.7 ± 2.2
starch	103.1 ± 3.5
povidone	97.8 ± 1.4
talc	96.6 ± 3.3
calcium phosphate	98.4 ± 4.7

### 3.10 Application of the QTP Determination Method in Pharmaceutical Tablets and Recovery Tests

The accuracy of the sensor and the possibility of matrix interferences were further investigated by using a recovery test. In the pharmaceutical analysis, amounts of QTP were added to samples, and the recovery percentage values were calculated from the actual and added QTP concentration. The QTP recovery percentage in the spiked samples ranged between 97.80 and 100.24% as shown in Table 6. These results indicated that QTP analysis can be carried out without interference from the tablet matrix. It is noticeably demonstrated that the proposed method is a feasible, sensitive, and efficient tool for QTP determination of pharmaceutical samples.

**Table 6:** Results of QTP Recovery Experiments with the Tablet Pharmaceutical Samples of Poly­(l-cys)/GCE Method

*C*_QTP_ added^a^	*C*_QTP_ expected^a^	*C*_QTP_ found^a^	recovery (%)
0.00		56.6	
11.20	67.80	66.88 (±0.95)	97.80 (±1.06)
18.20	74.80	74.93 (±1.52)	100.24 (±2.48)
28.40	85.00	84.52 (±1.58)	99.24 (±2.22)

a Concentration units: μmol L^–1^.

### 3.11 Electrochemical vs Spectrophotometric Methods for QTP Determination

The UV–vis spectrophotometric behavior of quetiapine (QTP) revealed two prominent absorption bands in the ultraviolet region with maxima at 254 and 294 nm. The peak observed at 254 nm corresponds to π → π* electronic transitions, whereas the absorption at 294 nm is attributed to n → π* transitions, both associated with the aromatic framework of the dibenzothiazepine ring system. The QTP UV spectra at different concentrations are shown in Figure 10.

**Figure 10:**
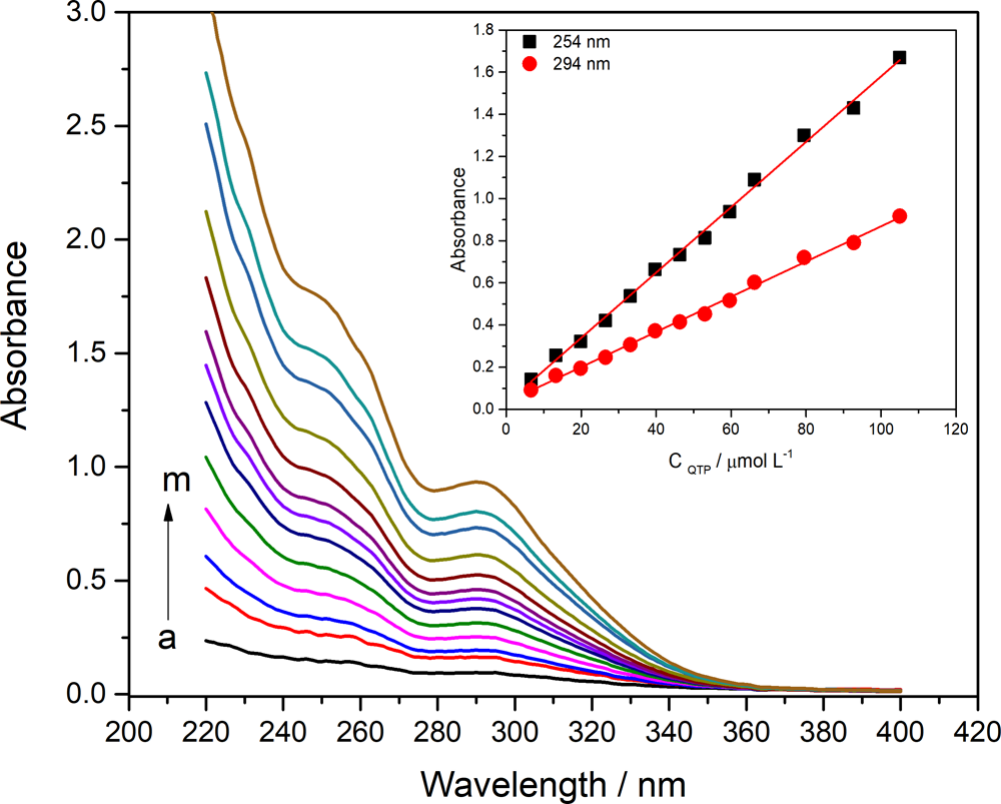
UV–vis spectra of the solutions of QTP. (a) 6.62, (b) 13.24, (c) 19.86, (d) 26.49, (e) 28.05, (f) 33.11, (g) 39.73, (h) 46.31, (i) 52.98, (j) 59.60, (k) 66.2, (l) 79.47, and (m) 92.72 μmol L^–1^. Inset: Absorbance vs *C*_QTP_.

The QTP concentrations were evaluated within the range of 6.62–92.72 μmol L^–^^1^, with absorbance measurements performed at two distinct wavelengths: 254 and 294 nm. In both cases, a linear relationship was observed between the absorbance and analyte concentration across the studied interval. These correlations are represented by eq 6 and eq 7, corresponding to each detection wavelength.

$$A_{254\;\mathrm{nm}}=-0.028(\pm 0.014)+0.015(\pm 0.002)C_{\mathrm{QTP}}/\mu \mathrm{mol}\;L^{-1\;},(R^{2}=0.997)$$ 6

$$A_{294\;\mathrm{nm}}=-0.037(\pm 0.007)+0.008(\pm 0.001)C_{\mathrm{QTP}}/\mu \mathrm{mol}\;L^{-1\;},(R^{2}=0.996)$$ 7

At 254 nm, the slope of the calibration curve was greater than that at 294 nm. Thus, for QTP determination, a wavelength of 254 nm was chosen for the comparison method because of its greater sensibility.

The poly­(l-cys)/GCE was used to determine QTP in tablets. Experiments were performed in triplicate, employing the standard addition approach. The QTP content in tablets was additionally determined using the official spectrophotometric method.^55^ A statistical comparison through the paired *t* test and Fisher’s exact test^56,57^ (Table 7) indicated no significant difference at a 95% confidence interval, confirming that the poly­(l-cys)/GCE sensor yields comparable results and can be successfully used for voltammetric determinations of QTP in tablets.

**Table 7:** QTP Determination Results in Tablets according to the Proposed Method and the Official Spectrophotometric Protocol

sample	label value (mg per tablet)	by the proposed method (mg per tablet)	by the official method (mg per tablet)
tablet	200	199 (±4.58)	193 (±3.09)

## 4 Conclusions

This study describes an l-cysteine electropolymerization application for the development of sensors for QTP pharmaceutical analysis. The sensor is a feasible alternative for QTP determination through SWV. The poly­(l-cys)/GCE sensor demonstrated an outstanding analytical performance for QTP quantification, likely stemming from minimal charge-transfer resistance and electrocatalytic enhancement. Optimized conditions enabled high sensitivity and detection limits comparable to those reported in previous electrochemical approaches. The method also exhibited good intra- and interday precision and offered the added benefits of simplicity, rapid preparation, and low cost.

## Supplementary Material



## Notes

(Figure S1) Capacitive current as a function of the scan rate, with linear fitting of the capacitive region used to determine differential capacitances and roughness factors; (Figure S2) relationship of the number of cycles of the electropolymerization procedure and reduction current at +0.7 V; (Figure S3) relationship of the number of cycles of the electropolymerization procedure and oxidation current of 1.0 mmol L^–1^ QTP (PDF)
