# Supplementary material for: Analytical Application of an l‑Cysteine-Based Electrochemical Sensor for Quetiapine Determination in Pharmaceuticals
Source: ACS Omega. 2025 Sep 30;10(40):47517–27. doi: 10.1021/acsomega.5c07368 (PMC12529160; doi:10.1021/acsomega.5c07368)
Supplement: Supplementary file 1 [file ao5c07368_si_002.pdf]

Analytical application of an L-Cysteine-Based electrochemical sensor for quetiapine determination in pharmaceuticals

Maria Eduarda C. Goulart [a], Lucas D. Paquini [b], Luciene P.R. Profeti [b], Demetrius Profeti [c], Bruno R. L. Ferraz [c*]

[a] Department of Pharmacy and Nutrition, Federal University of Espírito Santo UFES, 29.500-000, Alegre, ES, Brazil

[b] Laboratório de Pesquisa e Desenvolvimento em Eletroquímica, Federal University of Espírito Santo UFES, 29075-910 Vitória, ES, Brazil

[c] Department of Biology, Federal University of Espírito Santo UFES, 29.500-000, Alegre, ES, Brazil

* e-mail: bruno.ferraz@ufes.br

Supplementary informaton

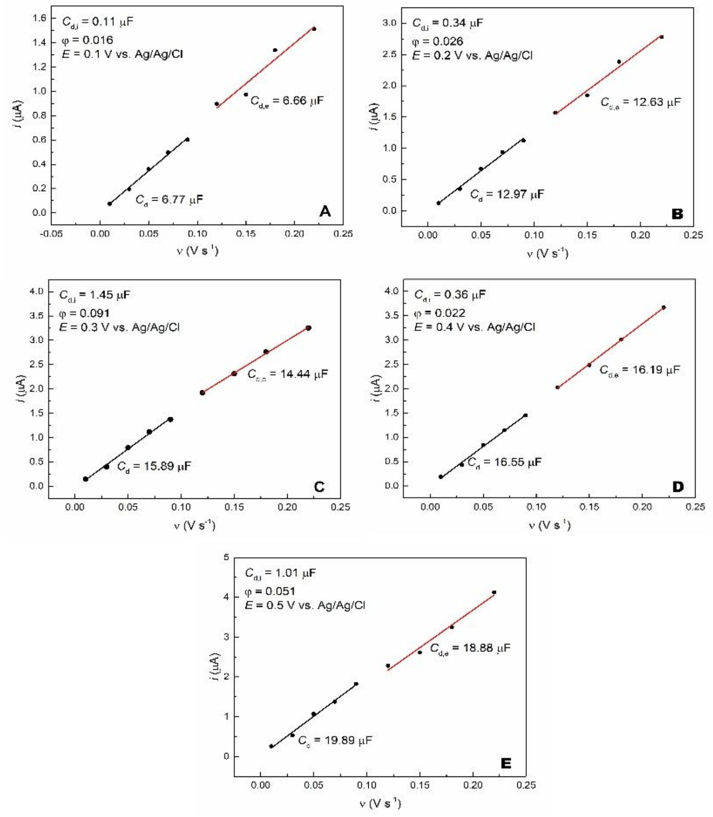

Figure S1. Capacitive current as a function of scan rate, with linear fitting of the capacitive region used to determine differential capacitances and roughness factors. Experimental conditions: potential window of 0.1 – 0.5 V vs. Ag/AgCl; KCl concentration = 0.1 mol L^{-1}; scan rate range = 10–220 mV s^{-1}.

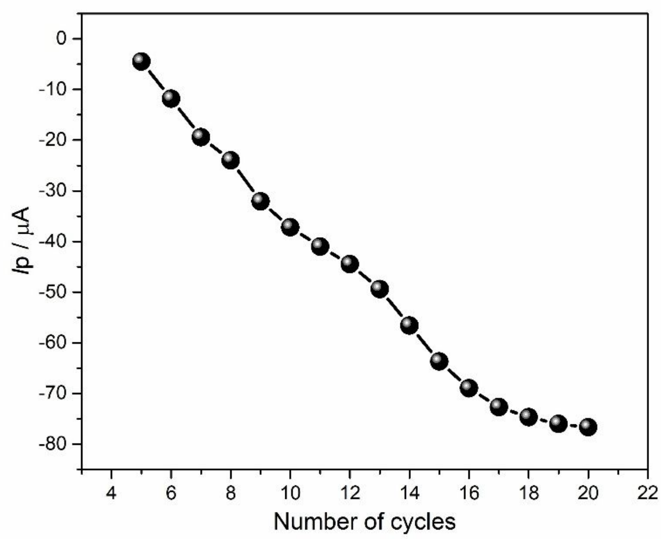

Figure S2. Relationship of number of cycles of electropolimerization procedure and reduction current at +0.7 V. C L-cysteine= 5.0 mmol L^{-1} in 0.10 mol L^{-1} of KCl solution, pH= 4.0, and υ = 100 mV s^{-1}.

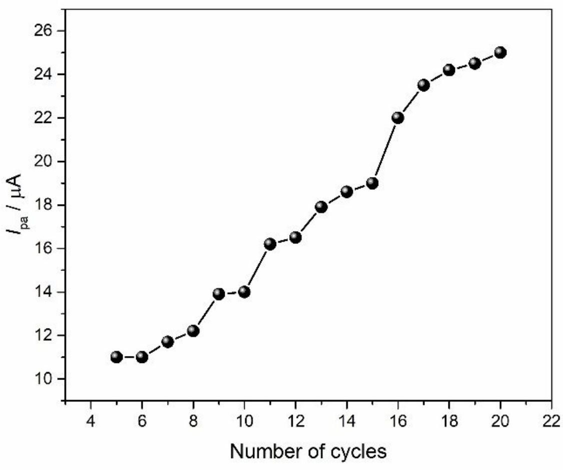

Figure S3. Relationship of number of cycles of electropolimerization procedure and oxidation current of 1.0 mmol L^{-1} of QTP. Electrolyte: 0.10 mol L^{-1} KCl (pH = 4.0).
